# Characterization of the virome associated with *Haemagogus* mosquitoes in Trinidad, West Indies

**DOI:** 10.1038/s41598-021-95842-6

**Published:** 2021-08-16

**Authors:** Renee Ali, Jayaraman Jayaraj, Azad Mohammed, Chinnadurai Chinnaraja, Christine V. F. Carrington, David W. Severson, Adesh Ramsubhag

**Affiliations:** 1 Department of Life Sciences, Faculty of Science and Technology, The University of the West Indies, St. Augustine Campus, St. Augustine, Trinidad and Tobago; 2 Department of Preclinical Sciences, Faculty of Medical Sciences, The University of the West Indies, St. Augustine Campus, St. Augustine, Trinidad and Tobago; 3 Department of Biological Sciences and Eck Institute for Global Health, University of Notre Dame, Notre Dame, IN USA; 4 Department of Medical and Molecular Genetics, Indiana University School of Medicine, South Bend, IN USA

**Keywords:** Microbiology, Molecular biology, Zoology

## Abstract

Currently, there are increasing concerns about the possibility of a new epidemic due to emerging reports of Mayaro virus (MAYV) fever outbreaks in areas of South and Central America. *Haemagogus* mosquitoes, the primary sylvan vectors of MAYV are poorly characterized and a better understanding of the mosquito’s viral transmission dynamics and interactions with MAYV and other microorganisms would be important in devising effective control strategies. In this study, a metatranscriptomic based approach was utilized to determine the prevalence of RNA viruses in field-caught mosquitoes morphologically identified as *Haemagogus janthinomys* from twelve (12) forest locations in Trinidad, West Indies. Known insect specific viruses including the Phasi Charoen-like and Humaiata-Tubiacanga virus dominated the virome of the mosquitoes throughout sampling locations while other viruses such as the avian leukosis virus, MAYV and several unclassified viruses had a narrower distribution. Additionally, assembled contigs from the Ecclesville location suggests the presence of a unique uncharacterized picorna-like virus. Mapping of RNA sequencing reads to reference mitochondrial sequences of potential feeding host animals showed hits against avian and rodent sequences, which putatively adds to the growing body of evidence of a potentially wide feeding host-range for the *Haemagogus* mosquito vector.

## Introduction

Mosquitoes serve as important vectors for many infectious agents that contribute to significant morbidity and greater than 1 million human deaths yearly^1,2^. Viruses transmitted by mosquito vectors include dengue virus, chikungunya virus, yellow fever, and Zika virus, which have all caused major pandemics within recent times. Furthermore, many neglected tropical and other emerging viral diseases transmitted by mosquitoes are still poorly understood. The advent of deep sequencing technologies has now made the detection and quantification of viral agents much easier and affordable. This technological advancement is now contributing to increased discovery rates and a better understanding of uncharacterized viruses, including many insect-associated viruses of human health importance^3–9^. Previously uncharacterized viruses with unknown pathogenicity against humans or wildlife have also been detected by RNA techniques^10,11^. Furthermore, microbiome-based analyses allow for the elucidation of interactions among viruses and other biological systems associated with mosquitoes and their effect on vector competence and transmission of infectious viral agents.

The mosquito *Haemagogus janthinomys* (*Hg. janthinomys*) has been reported as the primary vector in the transmission of the Mayaro virus (MAYV), an emerging alphavirus endemic to regions of South and Central America^12–15^. The recent detection of MAYV fever cases in previously unreported areas in South America has triggered alarm bells concerning the spread of this virus in the Americas^15–18^. Several epidemiological models predict this virus will follow the steps of the Zika and chikungunya viruses in causing a major epidemic in the future^19,20^. In the event of an epidemic, understanding the virus-host interactions would be critical for developing effective management systems for the MAYV disease. Furthermore, the role of urban mosquitoes such as *Aedes* and *Anopheles* species as a potential driving force for spread of MAYV has also been investigated^21,22^. However, *Haemagogus* species are the poorly characterized mosquitoes and there is little data available on their viral transmission dynamics.

In this study, we used a transcriptomic-based approach to map the RNA virome of wild caught adult female mosquitoes morphologically identified as *Hg. janthinomys* from twelve forested locations in Trinidad, West Indies.

## Results

### RNA Sequencing data from field caught *Haemagogus* mosquitoes

All mosquitoes from the twelve forested locations included in the RNA sequencing analysis were identified as *Hg. janthinomys* based on standard taxonomic keys^23–25^ and wing geometric morphology^26^. However, a subsequent barcoding study conducted on *Hg. janthinomys* from Trinidad based on sequencing of the cytochrome c oxidase subunit I (COI) gene and the internally transcribed spacer region 2 (ITS2) showed that the population had three distinct genotypes that may represent a species complex, but this needs to be confirmed by additional studies (Ali et al., unpublished data).

**Figure 1:**
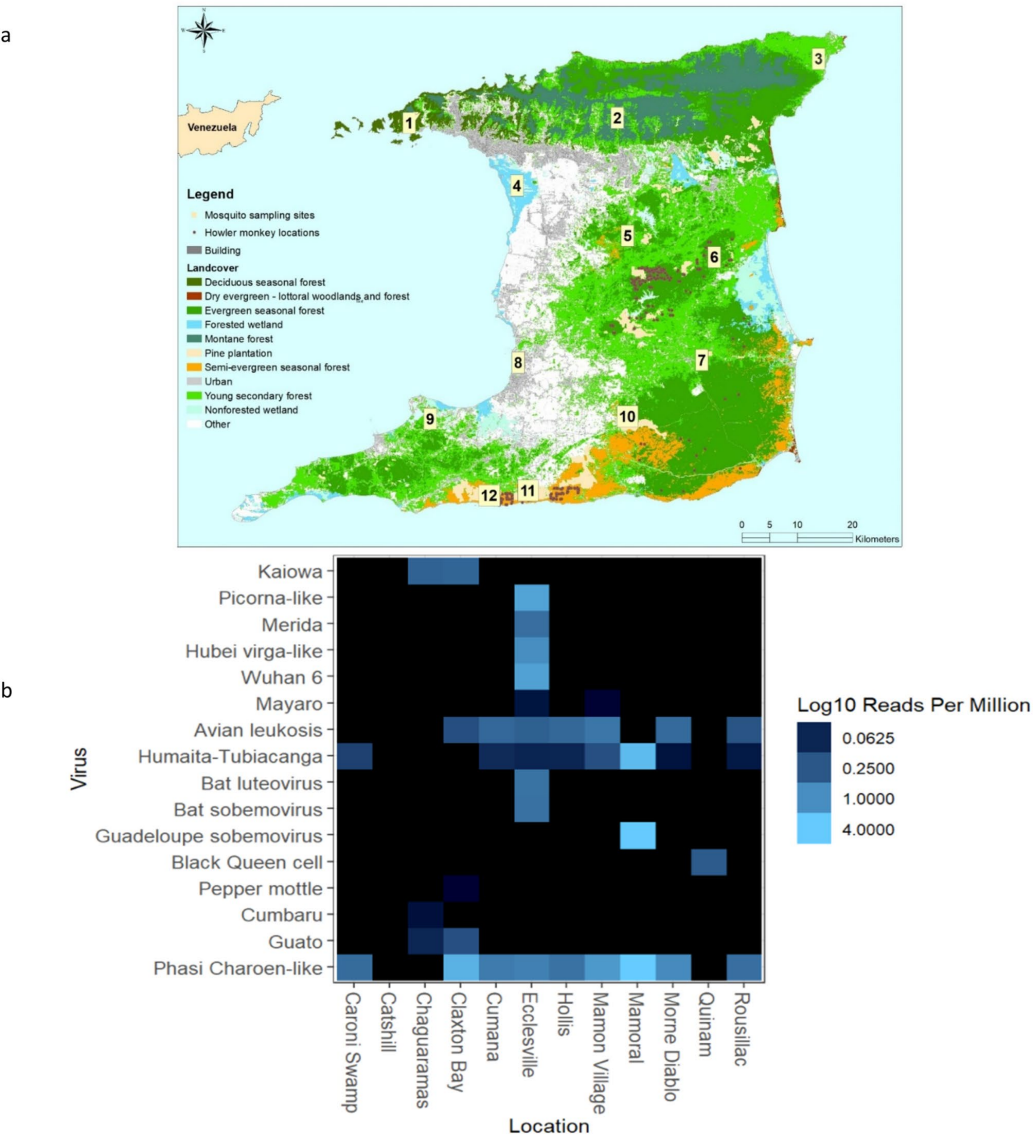
(**a**) *Haemagogus* mosquito sampling sites and (**b**) heatmap of viral reads from twelve forested locations in Trinidad. 1: Chaguaramas (CH), 2: Hollis (H), 3: Cumana (CU), 4: Caroni Swamp (CBS), 5: Mamoral (MO), 6: Mamon village (MA), 7: Ecclesville (E), 8: Claxton Bay (CB), 9: Rousillac (R), 10: Catshill (CA), 11: Morne Diablo (MD), 12: Quinam (Q).

Total RNA sequencing of the *Haemagagus* mosquito pools from the 12 sites yielded a total of 857,590,224 paired end reads after quality control processing, with an overall mean of 71,465,852 and a range of 57,884,090 to 87,768,438 reads as shown in Table 1. Direct Bowtie2 mapping of the total RNA sequences onto representative nucleic acid databases showed average mapping rates of 2415.39 reads per million (RPM) per location against reference viral sequences, 4.60 RPM against potential mosquito feeding host genomes and 1211.60 RPM against the Silva reference 16S bacterial database. The sites with the highest viral species richness were the Ecclesville and Mamon Village locations which included alphaviruses, insect-specific, mosquito-associated, and other viral types, with remaining collection sites having lower viral species richness (Fig. 1).

**Table 1:** RNA read mapping against a reference viral RVDB database, ARB/SILVA 16S rRNA gene sequences and potential animal feeding host reference mitochondrial sequences.

Location	Total number of reads	Number of reads mapped to viruses, bacteria and feeding hosts sequences	Unassigned Reads	Mapped reads per million (RPM)		
				Viral	Bacterial 16S	Feeding hosts
Chaguaramas	87,768,438	700,854	87,067,584	1.40	7966.13	17.78
Hollis	64,472,200	12,547	64,459,653	3.69	190.55	0.33
Cumana	65,560,414	21,746	65,538,668	4.84	326.40	0.46
Caroni Swamp	78,559,144	27,808	78,531,336	2.02	341.48	10.48
Mamoral	72,054,356	2,080,787	69,973,569	28,634.83	243.19	0.00
Mamon Village	65,353,074	54,579	65,298,495	21.74	811.55	1.85
Ecclesville	66,759,470	45,173	66,714,297	100.06	575.91	0.67
Claxton Bay	79,587,104	51,468	79,535,636	203.26	556.64	0.87
Rousillac	76,745,030	31,298	76,713,732	2.71	404.69	0.42
Catshill	65,843,962	349,318	65,494,644	0.00	2301.52	3.72
Morne Diablo	57,884,090	34,659	57,849,431	9.23	570.90	18.64
Quinam	77,002,942	19,329	76,983,613	0.90	250.12	0.00

### Viral sequences

Mamoral (28,634.83 RPM) overwhelmingly had the highest proportion of mapped viral reads followed by Claxton Bay (203.26 RPM) and Ecclesville (100.06 RPM) (Table 1). The other sites had relatively low proportions of viral reads (0.90–22.83 RMP) except for Catshill, where no viral read was detected. Generally, the majority of the viral RNA reads mapped to known insect specific viruses which included the Phasi Charoen-like and Humaita-Tubiacanga viruses. Additionally, RNA sequencing reads mapped to several viruses associated with a range of insects and feeding hosts and included some of human health importance. Trinity v2.9.1 assembly of mapped viral reads resulted in a total of 171 viral contigs and 4 unassembled reads (Table 2).

**Table 2:** Identification of viral contigs from *Haemagogus* mosquitoes collected in Trinidad.

	Virus of nearest match (% nucleotide identity)	Main reported host	Contig size range (nt)	Contig identity	# of contigs
**Mosquito borne viruses**					
Family; Togaviridae Alphavirus ssRNA(+)	Mayaro Virus (99.29–100%)	*Haemagogus spp*	149–277	Polyprotein gene, nsp1-nsp4	3^a^
**Known insect specific viruses**					
Genus; Phasivirus ssRNA(−)	Phasi Charoen-like virus (94–100%)	*Aedes aegypti*	243–6675	Glycoprotein, nucelocapsid gene	84
Unclassified ssRNA(+)	Humaita-Tubiacanga virus (97–99%)	*Aedes aegypti*	243–1998	Capsid, RNA dependant RNA polymerase, segment 1	14
**Mosquito associated viruses**					
Family; Bunyaviridae Phasmavirus like. ssRNA(−)	Wuhan mosquito virus 6 (95–97%)	Culex pools	2162	Complete cds	1
Unclassified	Kaiowa virus (99–100%)	*Culex & Stegomiya spp*	826–1172	Glycoprotein	2
Unclassified	Hubei virga-like virus 2 (94–95%)	*Culicidae spp*	547–5596	Hypothetical protein, RdRp & putative coat protein	3
Family Rhabdoviridae ssRNA(−)	Merida virus (91–95%)	*Culex & Ochlerotatus spp*	234–671	Nucleoprotein, glycoprotein,phoshphoprotein,RNA dependent RNA polymerase gene	10
Unclassified	Cumbaru virus (93%)	*Culicidae spp*	203	Glycoprotein	1
Unclassified	Guato virus (98–99%)	*Culex spp*	256–818	Hypothetical glycoprotein	5
Sobemovirus related ssRNA (+)	Guadeloupe mosquito virus strain (99.09%)	*Aedes aeqypti*	2978	Segment 1	1
Unclassified Picornavirales ssRNA (+)	Ecclesville picorna-like virus	No previous report	320–4519	Polyprotein gene	5

Classification	Virus or nearest match	Main reported host	Contig size range (nt)	Contig identity	# of contigs
**Other insect associated viruses**					
Genus: Cripavirus ssRNA(+)	Black Queen cell virus (96.02–97.14)	Apis spp	208–670	Capsid protein, nonstructural & polyprotein gene	5
**Other viruses**					
Genus: Alpharetrovirus ssRNA(RT)	Avian leukosis virus (99–100%)	Birds	217–1020	Envelope polyprotein, receptor binding protein, transmembrane envelope protein	33
Unclassified	Bat sobemovirus (93.79 – 95.36%)	Bats	289–321	Capsid protein	2
Unclassified	Bat luteovirus (95.46– 96.37%)	Bats	441–442	Capsid protein	2
**Plant viruses**					
Genus:Tobamovirus ssRNA(+)	Pepper mild mottle virus (98.68%)	*Capsicum* spp.	269	Coat protein, replicase	1

Assembly was performed on mapped sequencing reads from the twelve locations and identified using the blastn suite from the NCBI nucleotide collection nt/nr database.

^a^In addition to the 3 MAYV contigs from Ecclesville, there were also 4 unassembled MAYV reads from Mamon village.

The complete data set, which includes range of percentage nucleic acid identities and contig information, is shown in Table S1 and sequences are available at https://zenodo.org/record/4932469#.YMQbQKhKi01.

### Known Insect Specific Viruses (ISVs)

**Figure 2:**
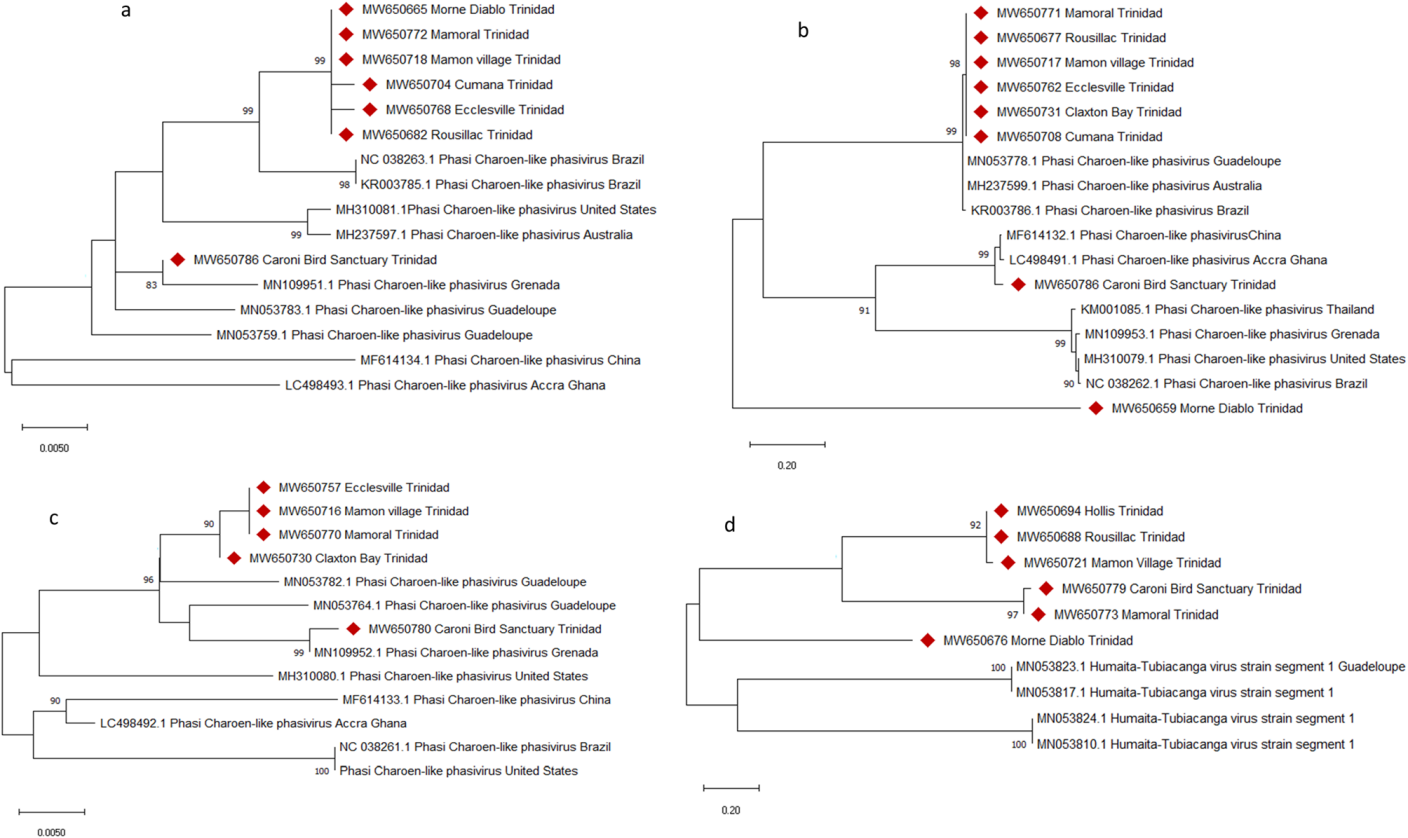
Maximum-likelihood phylogenetic trees of selected (**a**) Phasi Charoen-like segment S (551 bp), (**b**) Phasi Charoen-like segment L (451 bp), (**c**) Phasi Charoen—like segment M (439 bp) and (**d**) Humaita-Tubiacanga segment 1 (188 bp) of viral sequences from forest locations in Trinidad. The trees were constructed in MEGA X (Version 10.1), using the Kimura 2-parameter substitution model and 1000 bootstrap pseudoreplicates. Only bootstrap values ≥ 80% are shown. The Trinidad sequences are tagged with the red diamonds.

The metaviromes (Fig. S1) were dominated by one ISV, the Phasi Charoen-like virus (PCLV), which has been previously reported in *Aedes aegypti* (*Ae. aegypti*) mosquitoes^7,27,28^. Overall, PCLV was identified in nine locations (1.65–17 064.28 RPM, mean = 1442.02 RPM) while the Humaiata-Tubiacanga virus (HTV), another well-known ISV, was identified in eight (0.09–710.30 RPM, mean = 59.33 RPM) of the twelve locations sampled (Fig. S1). De novo assembly of the viral reads using Trinity v2.9.1 resulted in 3 to 27 contigs (243–6675 bp) of PCLV per site with similarity levels to reference sequences in GenBank ranging from 95.44 to 99.87%. Phylogenetic analysis of segments of the S (551 bp), L (451 bp) and M (439 bp) regions of representative sequences from the different sites showed most of the local sequences clustering into one main branch for all the segments with high bootstrap support (Fig. 2). However, the sequences for all three segments from the Caroni Bird Sanctuary clustered separately from other Trinidad sequences into well-supported clades (81–99% bootstrap support) including reference sequences (Fig. 2a,c), with segments S and M having closest links to sequences from Grenada. Additionally, the local sequences of segment L from Morne Diablo also separated and formed a separate clade (Fig. 2c; 100% bootstrap support).

Assembly of the HTV viral reads showed 1–3 contigs per site (243–1998 bp) with similarities to reference sequences in GenBank ranging from 97.80 to 99.13%. Phylogenetic analysis of a 188 bp region of segment 1 from the different sites showed all local sequences clustering separately from four references from Guadeloupe, with five (5) of the Trinidad sequences in one distinct sub-clade with 91% bootstrap support, the Caroni Bird Sanctuary and Mamoral sequences in a second strongly supported sub-clade (97% bootstrap support), and the Morne Diablo sequence on a weakly-supported branch (Fig. 2d).

### Mosquito-borne viruses

A relatively small proportion of viral reads (4.80 RPM) were classified as alphaviruses (*Togaviridae* family**)**, which accounted for approximately 0.0005% of all viral reads from the twelve locations (Fig. S1). Among these, 10 reads (4 from Mamon Village and 6 from Ecclesville at 0.06 and 0.10 RPM, respectively) mapped onto the genome of the MAYV. Trinity assembly of the mapped MAYV viral reads showed the six reads (three read pairs) from Ecclesville assembled into three contigs 166–277 bp long (Table 3) that aligned to different regions of reference MAYV genomes. However, the four MAYV reads from Mamon Village were unassembled. Blastn analysis revealed, all the contigs and reads had 99.29%-100% similarity to MAYV non-structural (nsp1-3 and nsp4) and structural polyprotein coding regions (Table S1). Most of these sequences had highest similarity to reference South American MAYV sequences, the majority of which were from Venezuela, Bolivia, and Peru (Table 3). Unassembled read M_MAYVF2 from Mamon Village had the highest similarity matches to reference MAYV sequences that originated from Trinidad, and these came from strains isolated in the 1950s, when the first infections by MAYV were reported.

**Table 3:** Assembled and unassembled reads of Mayaro virus from Ecclesville and Mamon village respectively.

Location	Sequence	Sequence type	Length (bp)	Blastn analysis				
				Highest % ID	Number of GenBank sequences with highest % ID	Country of origin of sequences	Representative GenBank sequence	Alignment region (nt position)
Ecclesville	E_MAYV1	Contig	251	99.20	7	Peru, Venezuela	KP842807.1	3451–3701
Ecclesville	E_MAYV2	Contig	277	99.28	4	Venezuela	KP842799.1	1314–1590
Ecclesville	E_MAYV3	Contig	166	100	18	Bolivia, Peru, Venezuela	MK573246.1	2158–2323
Mamon village	M_MAYVF1	Unassembled read	149	100	6	Venezuela	KP842799.1	2695–2843
Mamon village	M_MAYVR1	Unassembled read	149	100	14	Bolivia , Peru and Venezuela	KP842799.1	2545–2653
Mamon village	M_MAYVF2	Unassembled read	150	100	35	Bolivia, Brazil, French Guiana, Haiti, Peru, Trinidad and Tobago (1954, 1957), USA (Louisiana) and Venezuela	MK837006.1	6448–6597
Mamon village	M_MAYVR2	Unassembled read	150	100	6	Venezuela	KP842799.1	6221–6369

The MAYV sequences and reads are available at: https://zenodo.org/record/4932469#.YMQbQKhKi01.

### Mosquito-associated viruses

A relatively small number of reads mapped to reference genomes of mosquito-associated viruses (Fig. S1) including the Wuhan mosquito virus 6 (Bunyaviridae) (35.13 RPM), Hubei virga-like virus 2 (Rhabdoviridae) (8.84 RPM) and the Merida virus (Unclassified) (1.86 RPM). These mosquitoes-associated viruses were identified in the Ecclesville, Chaguaramas, Claxton Bay and Mamoral locations. Overall, the Ecclesville metavirome (Fig. S1) was affiliated with a higher number of mosquito-associated viral reads (90.31 RPM) and showed the greatest diversity when compared to other sampling locations including Claxton Bay (1.92 RPM) and Chaguaramas (1.40 RPM).

All mosquito-associated viral reads identified in the Chaguaramas (Fig. 1) and the Claxton Bay metavirome (Fig. S1) belonged to the unclassified viruses. The Guadeloupe mosquito virus sequence (Sobemovirus) was the only mosquito-associated sequence identified in the Mamoral location. Assembly of the mapped viral reads using Trinity v2.9.1 resulted in 1 contig (2162 bp) for the Wuhan mosquito 6 virus, 3 contigs (547–5596 bp) for Hubei virga-like virus, and 10 contigs (234–671 bp) for the Merida virus. Blastn analysis of the contigs of the three viruses showed highest identity of 96.85%, 99.14–99.63% and 97.17–99.15%, respectively, to GenBank reference sequences. Based on phylogenetic analysis for the segment 3 region for the Wuhan mosquito 6 virus (2133 bp), the local Ecclesville sequence diverged from the reference sequences from Australia (Fig. S1a). Furthermore, 1 contig (2978 bp) for the Guadeloupe mosquito virus was identified only in the Mamoral metavirome. Phylogenetic analysis of an 1859 bp region of segment 1 (Fig. S1b) showed the sequence separating from all reference sequences from Guadeloupe. However, it should be noted that bootstrap values were not significant for separation of the Trinidad viral sequences from reference sequences for Wuhan mosquito 6 and the Guadeloupe mosquito virus.

### Other viruses

RNA sequencing reads from Eccleville also mapped to the picorna-like virus (44.40 RPM) and reads from the Chaguaramas site mapped to unclassified viruses including Kaiowa virus (1.16 RPM), Cumabru-like virus (0.08 RPM) and Guato-like virus (0.16 RPM). The Kaiowa virus (1.30 RPM) and Guato-like virus (0.62 RPM) were also detected in mosquitoes from Claxton Bay. Additionally, reads mapped to other viruses including the avian leukosis virus in Ecclesville (1.05 RPM), Mamon Village (2.57 RPM), Claxton Bay (0.58 RPM), Cumana (1.40 RP), Hollis (1.43 RPM), Morne Diablo (1.57 RPM) and Rousillac (0.69 RPM); the black queen cell virus in Quinam (0.90 RPM); and the pepper mild mottle virus in Claxton Bay (0.06 RPM).

Trinity v2.9.1 assembly of the reads resulted in 2 contigs (826–1172 bp) of the unclassified Kaiowa virus (99 to 100% similarity levels) in the Claxton Bay and Chaguaramas sites, as well as 1 contig (203 bp) of the Cumbaru-like virus (similarity level = 93%) and 4 contigs (256–300 bp) of the Guato-like viruses (similarity level = 98–99%) from the Chaguaramas location (Table S1). Kaiowa and Guato viruses were previously found in mosquitoes from Brazil^29^.

Furthermore, 5 contigs (320–4519 nt) from Ecclesville were unique, with highest similarity levels (94.94–96.94%) to the Atrato picorna-like virus. Phylogenetic analysis of a 350 bp region of the polyprotein segment (Fig. S1c) resulted in all Ecclesville contigs forming a single clade that diverged from the GenBank reference Atrato picorna-like virus sequence. A relatively large number (33) of contigs (217–1020 nt) from 7 locations (Hollis, Cumana, Mamon Village, Ecclesville, Claxton Bay, Rousillac and Morne Diablo) aligned with high nucleotide identity (99–100%) to the avian leukosis virus strains from China. Maximum likelihood phylogeny of the avian leukosis viral contigs resulted in local sequences separating into two main clades with sequences from Cumana and Hollis clustering together (Fig. S1d) and sequences from Rousillac and Mamon village forming a strongly supported clade. This suggests the local avian leukosis viruses are highly diverse and may include unique lineages as compared to the reference sequences available in the GenBank database. Four contigs (289–442 bp) from the Ecclesville location aligned with 94–96% nucleotide identity to unclassified bat sobemoviruses and luteoviruses (Table S1).

### Potential feeding host sequences

Mapping of RNA reads to potential mosquito blood-meal host mitochondrial sequences showed that, although levels were relatively low, there were hits to the avian species, *Gallus gallus,* at four locations (Claxton Bay- 0.61 RPM, Mamon Village- 1.85 RPM, Rousillac-0.49 RPM, and Hollis-0.75 RPM) and to the rodent species, *Mus musculus,* at two locations (Chaguaramas-17.73 RPM and Catshill-3.72 RPM). Assembly of the avian reads resulted in 17 contigs (203–982 nt) with > 99% nucleotide identity to *Gallus gallus* and assembly of the rodent reads resulted in 25 contigs (240–966 nt) with > 99% identity to *Mus musculus* (Table S1). The contig sequences are available at: https://zenodo.org/record/4932469#.YMQbQKhKi01.

### Bacterial sequences

**Figure 3:**
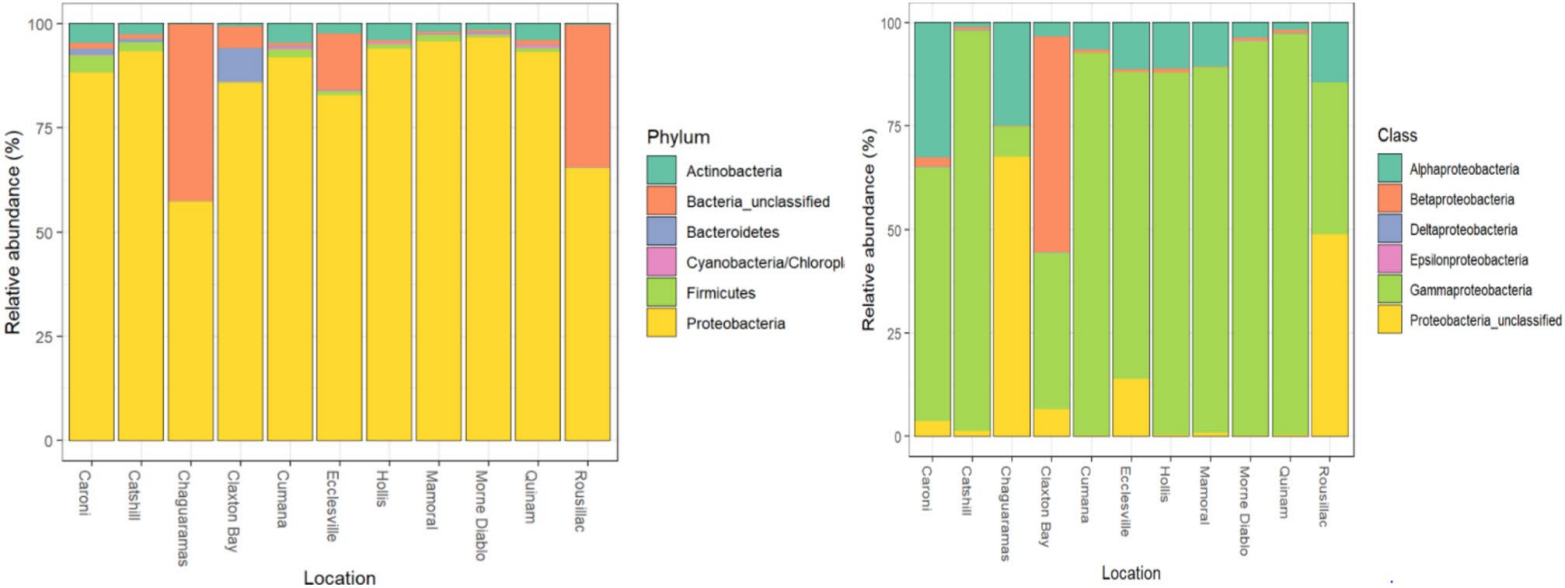
Plots of relative abundance of *Haemagogus* RNA sequence reads mapped to the Silva bacterial database showing phyla diversity and domination of Proteobacteria throughout all locations.

In all twelve locations, a relatively moderate level of reads (717.28 RPM) mapped to the SILVA bacterial 16S rRNA gene sequence database (Version 132). As seen in Fig. 3, the majority of mapped reads from all locations were classified as Proteobacteria (relative abundance = 96.5–57.3%), and within this phylum, the Gammaproteobacteria class dominated at all locations (relative abundance = 61.6–97.0%) except Chaguaramas (7.4%), Claxton Bay (37.9%) and Rousillac (36.6%). Mapping of the RNA sequences against 301 representative 16S rRNA gene sequences of *Wolbachia* spp. showed a mapping rate ranging from 0.92 Log10 RMP to 3.6 Log10 RPM. Assembly of reads mapped to the Wolbachia 16S rRNA genes resulted in 7 contigs (234–1569 nt) from four sites (Claxton Bay, Chaguaramas, Ecclesville, Rousillac) that showed 96.04–100% similarity to *Wolbachia* species based on Blastn analysis. The *Wolbachia* contig sequences are available at https://zenodo.org/record/4932469#.YMQbQKhKi01.

## Discussion

This is the first report on the virome of any *Haemagogus* mosquito species and provides important baseline data on a range of viruses associated with these mosquitoes. The data shows the presence of nucleic acid sequences from arboviruses of human and animal health significance in *Haemagogus* mosquitoes found in Trinidad, which may have epidemiological implications for this region. Viral sequences identified include those that belong to the families *Bunyaviridae*, *Togaviridae* and *Rhabdoviridae,* which have been previously reported to be a part of the viromes of other mosquito species^3–6,11^. The insect specific viruses (ISVs) characterized were generally similar for most of the sampling locations on the island apart from Catshill and Chaguaramas, where no ISV’s were identified. PCLV and HTV, the two most common ISVs, have also been identified in similar studies associated with field-caught *Ae. aegypti* mosquitoes from the Caribbean region as well as in Asia and Australia^7,28,30,31^.

Although, the average read depth for the two ISV’s were relatively low in comparison to *Ae. aegypti* from other studies, this is the first report of ISV’s identified in *Haemagogus* mosquito species. Furthermore, the broad distribution of both viruses in *Haemagogus* mosquitoes collected from the majority of sites was similar to observations of other researchers working with in field caught *Ae aegypti* mosquitoes^7,30^. This suggests that these ISVs may be naturally abundant and have a broad mosquito host range in the wild. However, there is need for further characterization of ISV’s from other mosquito species in the wild. The phylogenetic analyses showed relatively distinct sequences for all PCLV segments from Trinidad, except for the sequences from the Caroni Bird Sanctuary which clustered with the reference sequence from Grenada, an island close to Trinidad. This suggests a broad regional distribution for at least some of the PCLV viral lineages. Maximum likelihood phylogeny also showed sequences of Trinidad HTV as a distinct lineage that was separate from reference sequences from Guadeloupe. Due to the limited sample size and the fact that the study was only limited to the island of Trinidad, it is not possible to conclude whether the distinct lineages of viral sequences found were due to evolutionary processes in an isolated ecosystem. Additionally, several reports have shown that viral heterogeneity can be influenced by vector and host interactions^19,32^ but the potential role of these types of interactions on the occurrence of distinct clades of the PCLV and HTV sequences in Trinidad needs to be established.

The RNA sequence data from this study shows that the ISVs found in *Haemagogus* were similar to those reported in other Culicidae vectors and hence they may be broadly associated with mosquitoes. Furthermore, although this report has revealed ISVs known to be associated with other competent disease vectors, there is need for further characterization of ISV’s from other mosquito species in the wild.

MAYV was only identified at very low levels in the Ecclesville and Mamon Village locations. This finding was similar to previous studies which also reported isolation of the virus at low infection rates in pools of field caught *Hg. janthinomys* mosquitoes from South America^33–35^. Ecclesville and Mamon Village sites are in areas known to have primate troops (the Red Howler Monkey, *Alouatta macconnelli*), which are the main reservoir and host feeding preference reported for *Haemagogus* mosquitoes in the sylvatic cycle of this emerging alphavirus^13,16,19,35^. Hence, the non-human primates could be the possible source of the MAYV in *Haemagogus* from these sites. Most of the other sampling locations are not associated with non-human primates, which could have accounted for the absence of MAYV. There is limited information on the prevalence of MAYV in mosquitoes or potential hosts present in the island, but it must be noted that the first report of MAYV infections included four out of five patients who were forest workers in Trinidad, and three of these individuals were stationed at Catshill and Moruga sites that are relatively close to Ecclesville and Mamon Village^36^. However, the possibility of other feeding hosts at Ecclesville and Mamon Village sites being the source of MAYV also cannot be discounted since avian and small mammalian species have also been reported to be associated with MAYV isolation in previous studies^37,38^. *Haemagogus* mosquitoes have been shown to be very attracted to humans as a blood feeding source^33,35,39^ and have become well adapted to breeding in artificial containers^40–42^. The forest peripherals in Mamon Village and Ecclesville are used for housing and agricultural activities^42^ where humans may also serve as potential intermediary host when MAYV is in circulation.

RNA sequencing of the *Haemagogus* mosquito pool from Chaguaramas resulted in detection of unclassified Kaiowa, Guato and Cumbaru viruses (Fig. 1a) that were recently isolated from the salivary glands in *Culex* and other medically important mosquito species^29,43^. Additionally, other mosquito-associated viral sequences were identified in the Ecclesville location including a picorna-like virus, Merida virus and Hubei-virga like 2 virus. The Merida virus was previously initially isolated from the Yucatan’s capital state^44^ and Hubei virga like 2 from Hubei region in China^45^ which confirms a wide distribution and multiple Culicidae hosts for these viruses.

A unique unclassified picorna-like virus (Ecclesville picorna like virus) was also identified from Ecclesville, with the Atrato picorna-like virus 1 (Table S1) isolated from field *Psorophora* species along the Columbian river bank^46^ as its closest relative. Potential new lineages of the Wuhan mosquito virus 6 were also identified in Ecclesville whose location is ecologically disparate to the Hubei, China location where the virus was initially isolated from *Culex* mosquito species^47^. The Guadeloupe mosquito virus lineage (*Sobemovirus*) was also found to be associated with *Haemagogus* mosquitoes from Mamoral only, which suggests this virus may be regionally distributed and may have multiple mosquito hosts occupying different ecosystems since it was previously identified from urban *Aedes aegypti* collected from households in Guadeloupe^30^.

The findings of this study also showed the association of other viruses with *Haemagogus* that have not been reported before in any other mosquito species. The Pepper mild mottle virus was the only plant virus identified in this study and was found in the Claxton Bay *Haemagogus* mosquito pool. Culicidae mosquitoes are known to feed on nectar, plant fluids and fruit sap^48,49^, hence the uptake of plant associated viruses is not surprising and has also been reported in previous metagenomic mosquito studies^5–7,50^. The Pepper mild mottle virus is a capsicum virus^51^ known to be affiliated with insects such as aphids^49,52^, but this is the first record of the virus being associated with a mosquito species. Similarly, small numbers of viral reads were identified as Black queen cell virus (BQCV) from the Quinam location which may have been due to acquisition from environmental sources. Mosquitoes require carbohydrates as an energy source^53^ and nectar and plant secretions contains glucose and fructose sugars which are attractive to mosquitoes. Some *Haemagogus* mosquitoes may have fed on nectar or sugar exudates from plant flowers that infected bees may have previously fed on, resulting in the mosquito ingesting the virus. This insect associated virus has only been reported to be infective to honeybees around the world, including Australia and parts of South Africa^54,55^. This is the first study reporting the association of the BQCV with *Haemagogus* or any mosquito species. Further, this virus has not been previously reported in Trinidad and Tobago. The apiary industry has been in decline in the country due to the poor health status of hives^56^ and the role of the BQCV in this decline needs to be investigated.

The avian leukosis virus^57^ was also found throughout several sampling sites. This retrovirus is known to be associated with birds and is vertically transmitted (adult to baby chicks)^58^. Although some strains (Type E) are known to be endogenous in the genomes of birds, the sequences obtained in this study were highly similar to exogenous strains (Type J and RSA), suggesting that the mosquitoes may be acquiring the virus from feeding on host animals. The detection of this virus in *Haemagogus* from several locations strongly suggests birds as potential feeding hosts for these mosquitoes*.* Previous studies have shown that a mosquito’s blood-meal can provide evidence of potential feeding hosts using RNA-Seq data analysis^7,59^. Birds as potential feeding hosts for *Haemagogus* is further supported by the fact that bird sequences were the most common hits when RNA reads were mapped against common animals from Trinidad. Birds have also been previously suggested to be feeding hosts for *Haemagogus* in the MAYV sylvatic cycle^33,60^.

*Mus musculus* RNA was also identified in *Haemagogus* samples collected from Chaguaramas and Catshill locations suggesting rodents as an alternative feeding host for these mosquitoes. A previous entomological survey^42^ found *Haemagogus* mosquitoes present in high densities at locations in Trinidad with no known non-human primates, the reported major host of these mosquitoes^13–15^. Hence, the data from the current study supports previous suggestion that other animals may be serving as feeding hosts^61,62^. Some of the alternative hosts may also be potentially involved in the MAYV sylvatic cycle or may, in time, adapt to serve as reservoirs, which has major epidemiological implications for MAYV fever. Although MAYV has been detected in non-primate animals including birds, rodents and reptiles^33,37,60,63^, the ability of these animals to serve as strong reservoirs of the virus is not known and currently primates are still considered the primary reservoir^15,16,37^. Future outbreaks can be more widespread if the virus evolves to utilize animals like birds or rodents as efficient reservoirs since these animals are present within or are close to major human population centers. Epidemics can be further exacerbated by the widespread occurrence of *Haemagogus* mosquitoes, including in areas close to human communities in some countries like Trinidad and Tobago, as was noted by Ali et al. (2019).

This study is also the first report on the microbiome of the *Haemagogus* mosquito species inferred from the RNA-seq data. Although the Ribo-ZeroTM Magnetic kit (Illumina Inc.) was used to deplete ribosomal RNA, the depletion was evidently incomplete since a significant number of reads mapped to the Silva bacterial 16S rRNA database that showed dominance of the Gammaproteobacteria class within the Proteobacteria phylum. The high prevalence of this bacterial class has similarly been reported in other mosquito species including *Culex* and *Anopheles*^64–68^. More importantly, mapping against representative *Wolbachia* 16S rRNA gene sequences provided strong evidence for the association of this bacterial genus with *Haemagogus.* The presence of *Wolbachia* species is known to negatively affect insect vector competence, as was demonstrated for *Aedes* mosquito species transmission of mosquito-borne pathogens such as the Zika virus and dengue virus^67^. Additional studies are needed to determine if members of this bacterial genus may have similar effects in reducing vector competence of *Haemagogus* and transmissibility of MAYV and other *Haemagogus* transmitted arboviruses.

The lack of availability of whole genome sequences for any *Haemagogus* mosquito species made data analysis challenging since it was not possible to filter out the host genome sequences before analysis of sequences of taxonomic importance. Despite the challenges, this study has added valuable baseline data on *Haemagogus spp.* and highlights the need for further work on characterizing the virome and microbiome of these medically important mosquitoes.

## Methods

### Mosquito collection

Adult female mosquitoes were collected in twelve forested areas in Trinidad previously described by Ali et al. 2019 (Fig. 1) during the rainy season over the period June to December 2018 using the human bait adult catching method at ground level^69^. *Haemagogus* mosquitoes (n = 205) were collected and transported to the lab on dry ice. The specimens were morphologically identified using taxonomic keys^23–25^, pooled (3–5 mosquitoes) and stored at − 80 °C until further use. Mosquitoes with distinctive *Hg. janthinomys* morphological features were pooled and used for RNA sequencing. Subsequent to RNA sequencing, DNA bar-coding analysis of mosquitoes from Trinidad based on sequencing of the mitochondrial *cytochrome oxidase I* (COI) gene and the ITS2 region showed major sequence variations in these genes that suggest the possibility of the occurrence of a species complex (Ali et al., unpublished data). The mosquitoes analyzed in this study were subsequently referred to as *Haemagogus* spp.

### Nucleic acid preparation and sequencing

Mosquito pools were homogenized in 1 ml TRIzol LS reagent (Invitrogen) using a handheld cordless tissue homogenizer (VWR). Total RNA was extracted using the TRIzol LS / mosquito homogenate according to the manufacturers’ instructions. The RNA samples from the twelve locations were purified using the RNeasy MinElute Cleanup Kit (Qiagen) and shipped to Novogene Corporation Inc (Sacramento, California, U.S.A) for library construction and sequencing. The Ribo-ZeroTM Magnetic kit (Illumina Inc.) was used to deplete the ribosomal RNA. Libraries were sequenced from both ends (150 bp) on an Illumina platform (Illumina Inc, San Diego, U.S.A.) and the lncRNA pipeline was used to extract 150 bp paired end reads into FASTQ files.

### Viral read and contig analysis

**Figure 4:**
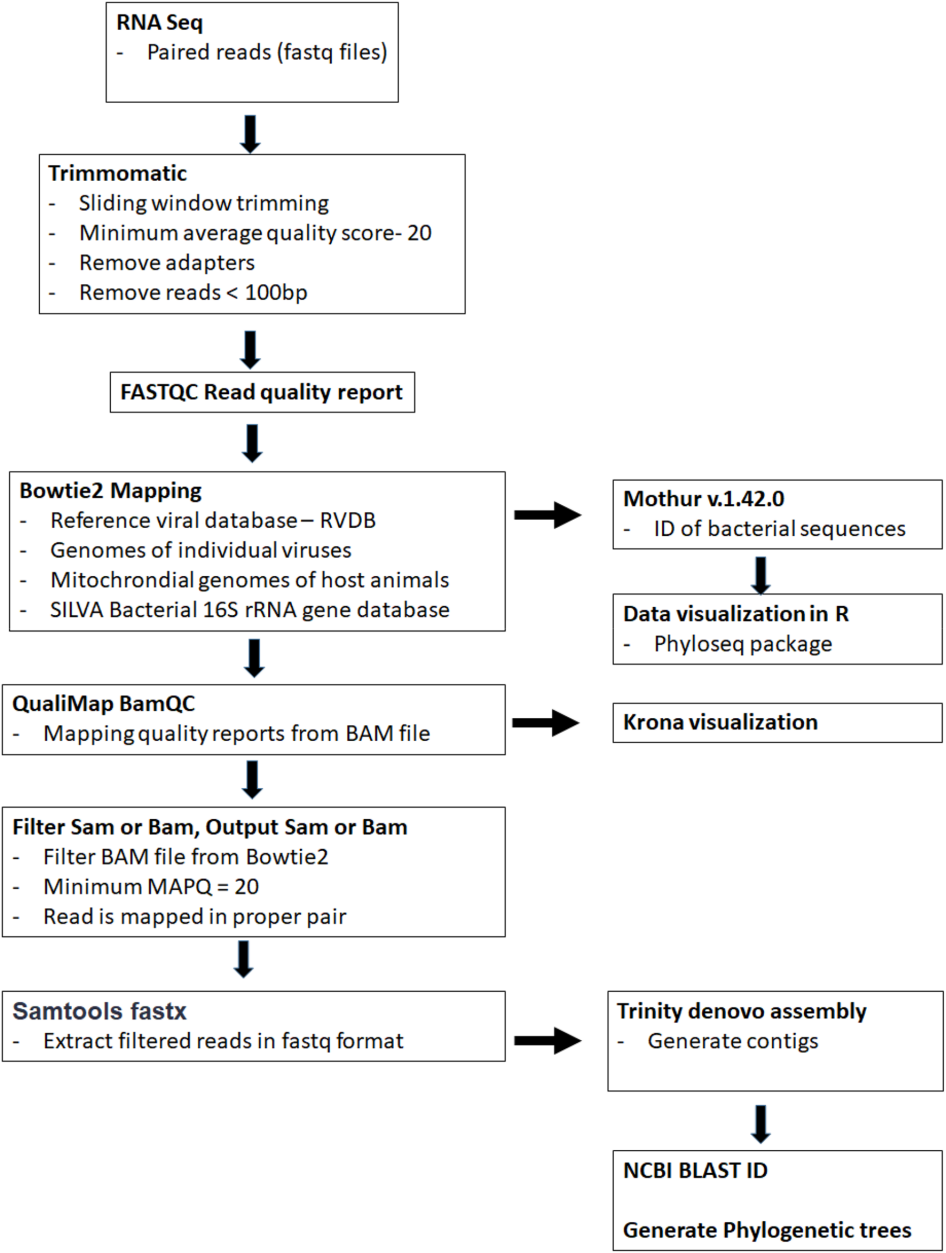
Workflow for bioinformatic analysis for viral, bacteria and feeding host read and contig analysis. All analyses were conducted using the Galaxy server (https://usegalaxy.eu/) except NCBI BLAST and generation of phylogenetic trees.

See Fig. 4 for workflow of bioinformatic analysis used to analyze the Illumina sequencing data. Host filtering was not conducted for contig generation since there is no reference genome available for *Haemagogus*. Raw paired-end reads were quality filtered (Q < 20) and trimmed for adaptor sequences in the Galaxy platform^70^ (www.usegalaxy.org) using Trimmomatic v0.36.6^71^. FastQC reports were generated for both paired files using FastQC v0.72^72^; the paired end reads were mapped against the RVDB: Reference Viral Database^73^ for a broader exploratory search and then against single reference genomes using Bowtie 2 v2.3.4.2^74^. Using BAM files generated from Bowtie 2 mapping from single reference genomes, Qualimap BamQC reports were generated using Qualimap BamQC v2.2.2d^75^. The BAM files generated from the Bowtie2 step were further filtered to a minimum MAPQ quality score of 20 using Filter SAM or BAM tool v1.1.1^76^ and the filtered reads were then extracted as FASTQ files using SAMtools fastx files v1.9^76^ for contig assembly. Trinity v2.9.1^77^ was used for de novo assembly of the generated FASTQ reads. Nucleotide similarity of viral contigs to published sequences (nucleotide collection nt/nr) was determined using the blastn suite v2.10.0^78^ from NCBI (www.ncbi.nlm.nih.gov). The distribution of viral identified reads was visualized using the Krona tool^79^.

### Bacterial read and contig analysis

Using the reference ARB/SILVA Version v138 SSU database^80–82^, ribosomal RNA sequences were mapped and extracted using the Bowtie 2 software v2.3.4.2^74^. The identity of reads was determined using mothur v.1.42.0^83^ after aligning to the mothur formatted RDP database (https://rdp.cme.msu.edu/) trainset16. Data visualization was done using the phyloseq package (v1.34.0) in R. The RNA sequences were further mapped directly onto 301 reference *Wolbachia* 16S rRNA gene sequences downloaded from the ARB/Silva ribosomal database (https://www.arb-silva.de/). The mapped reads were then assembled using Trinity (Galaxy version v2.9.1) and contigs generated were identified by Blastn.

### Feeding host read and contig analysis

Trimmed and filtered reads were mapped against 142 sequences of mitochondrial bar-coding genes and three full mitochondrial genomes of 116 animals using the Bowtie 2 software v2.3.4.2^74^ (Table S1). The animals included were 27 mammals, 10 reptiles and 79 bird species which are commonly found in Trinidad^84^. The sequences used for mapping are available at https://zenodo.org/record/4932469#.YMQbQKhKi01.

### Relative abundance of taxonomic groups

The relative abundance of the different taxonomic groups was expressed in reads per million (RPM) of total number of filtered reads per sample. The RPM was calculated using the formula:

$$RPM= No. \; of \; reads \; of \; taxonomic \; group \times \frac{\text{1,000,000}}{Total \; number \; of \; filtered \; reads \; in \; sample}$$

### Phylogenetic analysis

The phylogenetic relationships of selected viral assembled viral contigs and representative reference sequences from GenBank were determined using the MEGA X 10.0.5 software^85^. The contigs of all coding regions were aligned with the closest reference sequence from GenBank and amino acid sequences generated to confirm accuracy of the nucleotide sequences. Sequences were aligned using Clustal W and trimmed before phylogenetic trees were generated using the Maximum Likelihood method (Kimura 2-parameter substitution model) with 1000 bootstrap replications.

## Supplementary Information

Supplementary Information.

## Data Availability

The datasets analyzed during this study are available on the NCBI GenBank SRA database accession PRJNA689580 (https://www.ncbi.nlm.nih.gov/sra/PRJNA689580). The sequences are available in the NCBI GenBank database with accession numbers MW650653-MW650790, MW727408-MW727425, MW773204-MW773213 and MW779452-MW779457 (Table S1). All the sequence data generated during this study are also available at: https://zenodo.org/record/4932469#.YMQbQKhKi01.
