# Supplementary material for: Characterization of the virome associated with Haemagogus mosquitoes in Trinidad, West Indies
Source: Sci Rep. 2021 Aug 16;11:16584. doi: 10.1038/s41598-021-95842-6 (PMC8368243; doi:10.1038/s41598-021-95842-6)
Supplement: Supplementary file 1 — Supplementary Information. [file 41598_2021_95842_MOESM1_ESM.docx]

**Supplementary information for:**

Characterization of the virome associated with the Mayaro virus vector, *Haemagogus janthinomys*.

Renee Ali^1^, Jayaraman Jayaraj ^1^, Azad Mohammed^1^, Chinnadurai Chinnaraja^1^, Christine Carrington^2^, David W. Severson ^1,3^, Adesh Ramsubhag^1*^

^1^ Department of Life Sciences, Faculty of Science & Technology, The University of the West Indies, St. Augustine Campus, Trinidad and Tobago

^2^ Department of Pre-Clinical Sciences, Faculty of Medical Sciences, The University of the West Indies, St. Augustine Campus, Trinidad and Tobago

^3^ Department of Biological Sciences and Eck Institute for Global Health, University of Notre Dame, Notre Dame, Indiana, United States of America

* Corresponding author: [Adesh.Ramsubhag@sta.uwi.edu](mailto:Adesh.Ramsubhag@sta.uwi.edu)


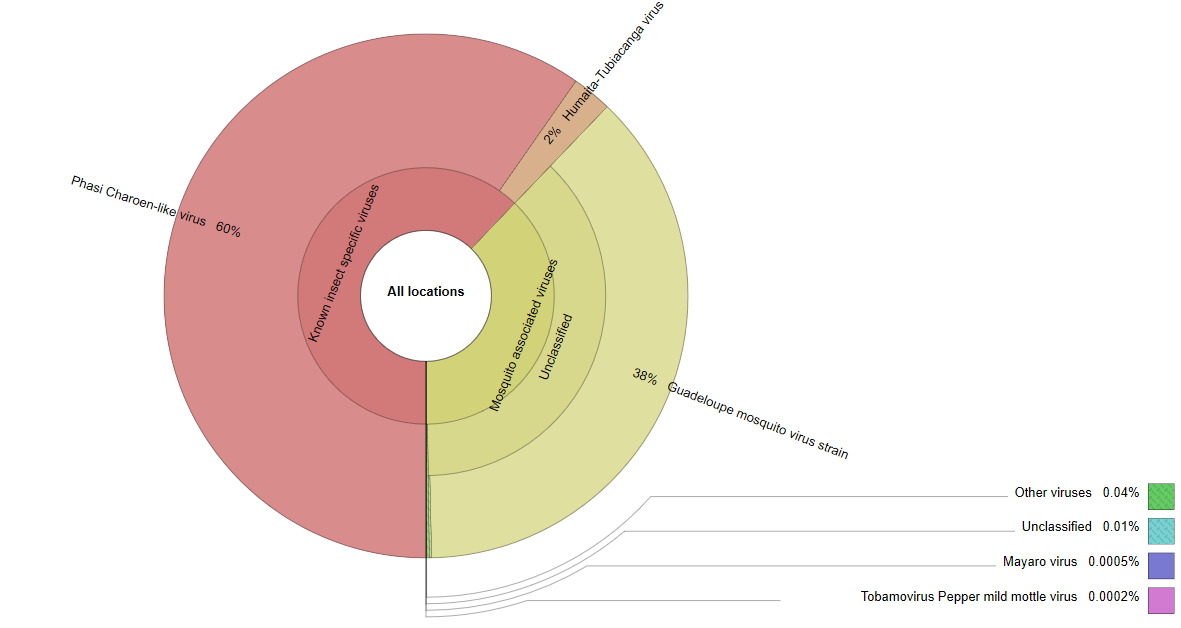


Fig. S1. Metavirome of *Haemagogus* mosquito vector from twelve forest locations in Trinidad. The Krona chart represents reads (n =2086764) mapped onto genomes of major viral groups.


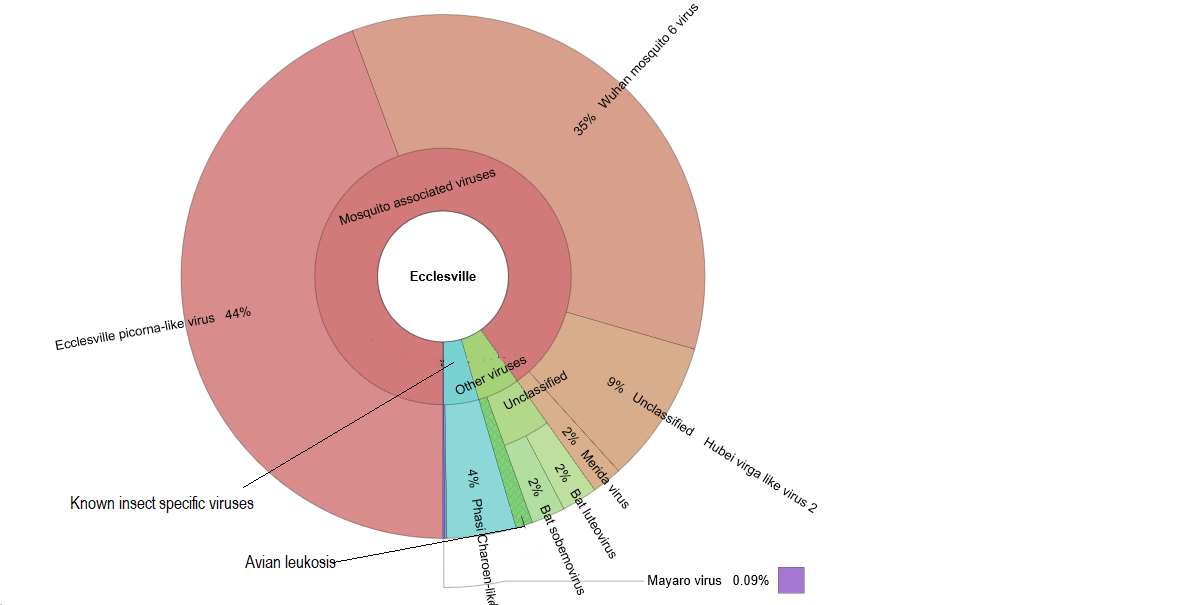


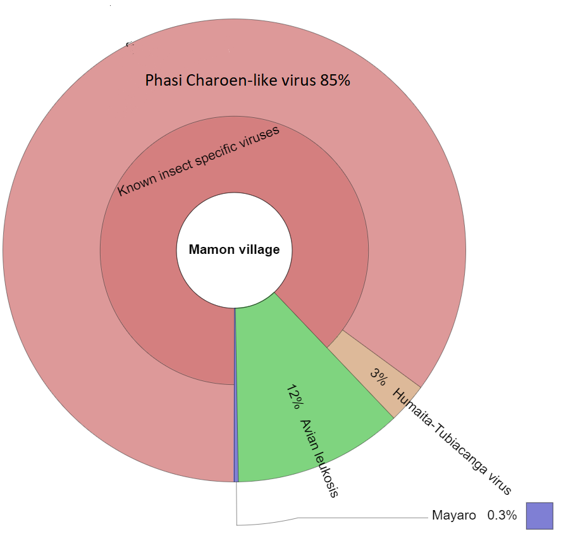


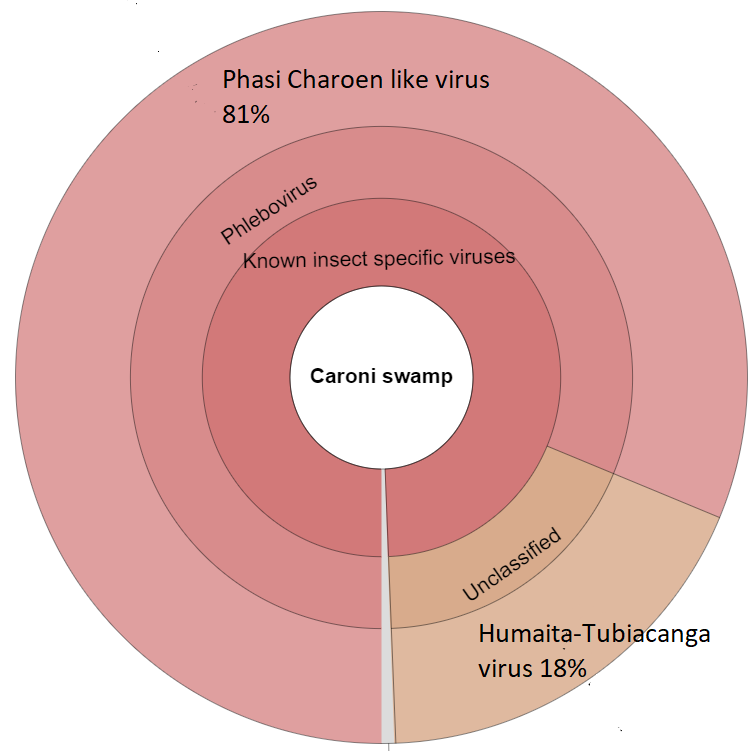

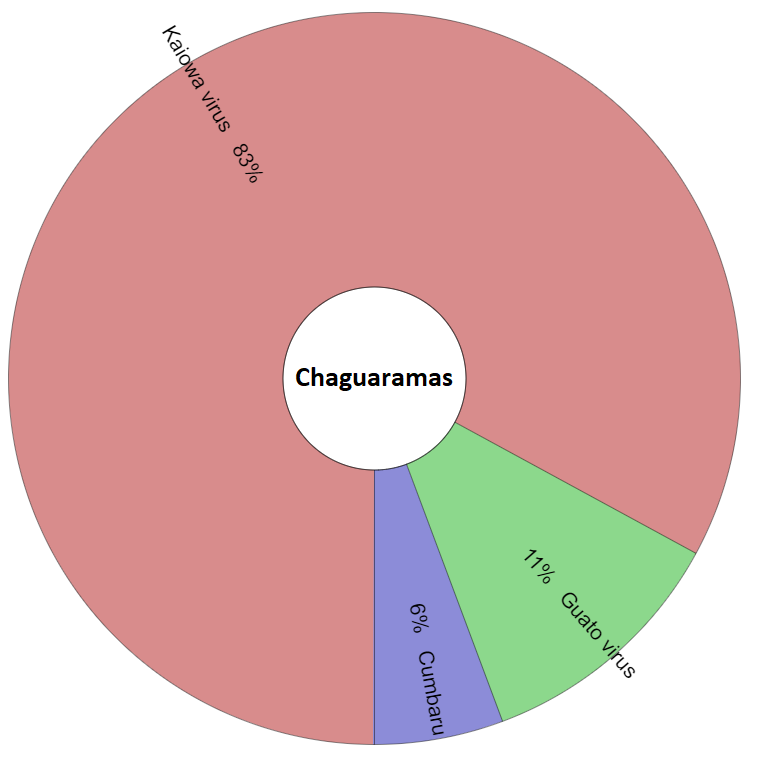


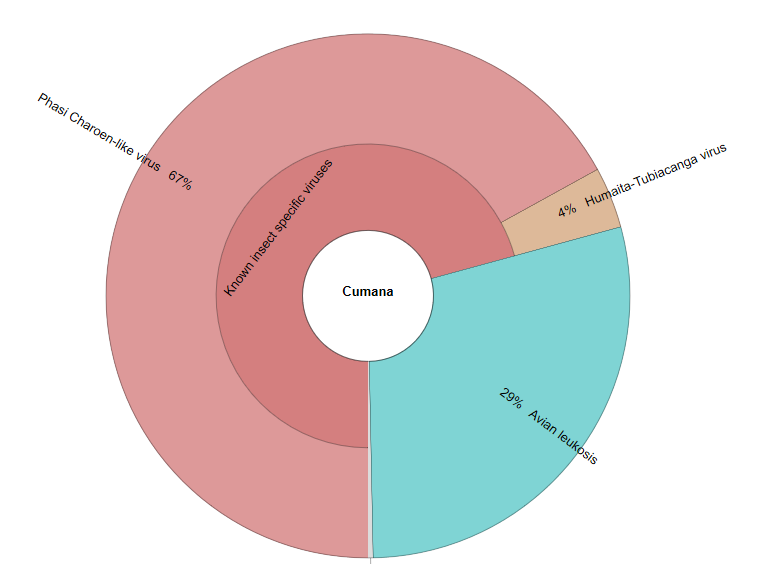

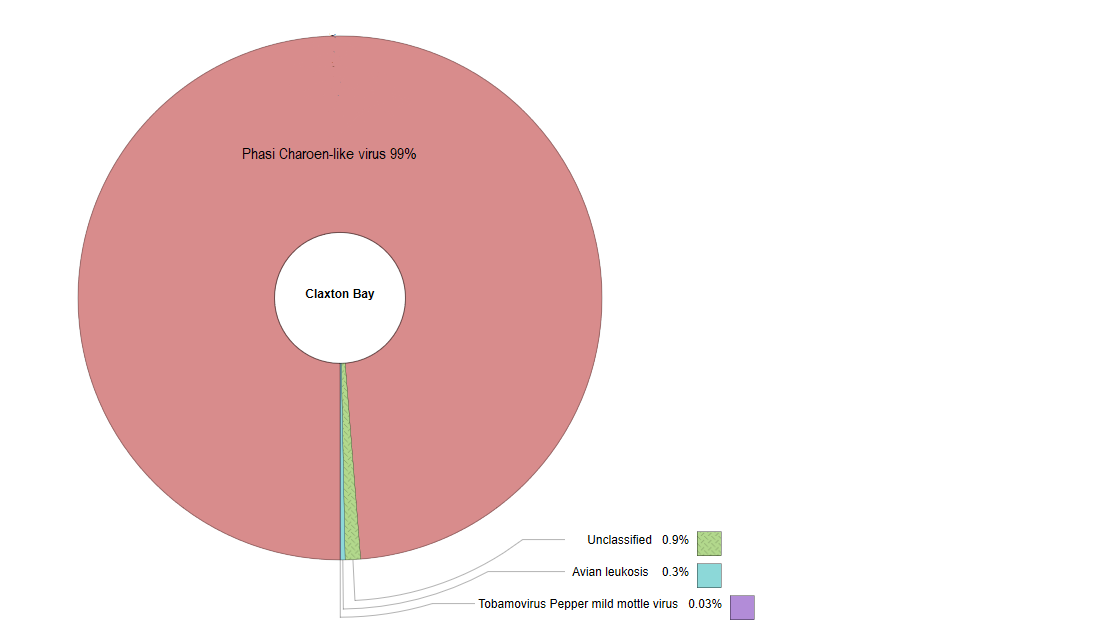


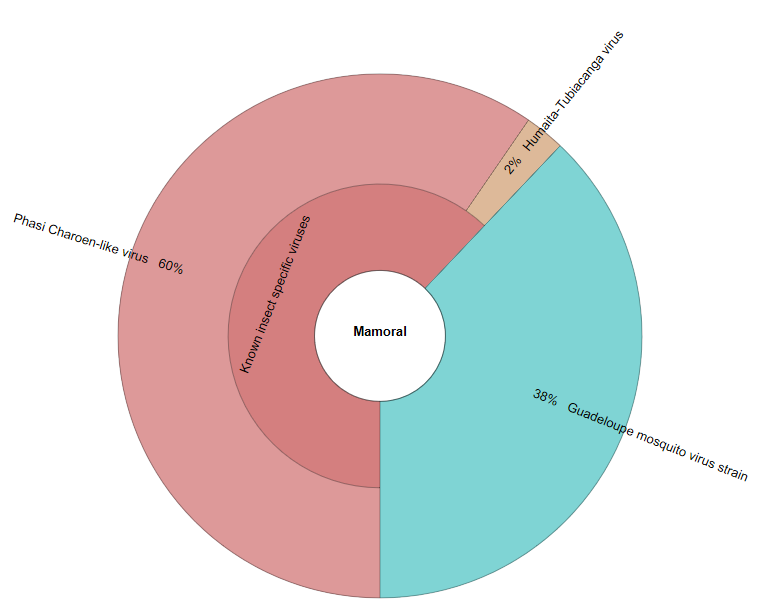

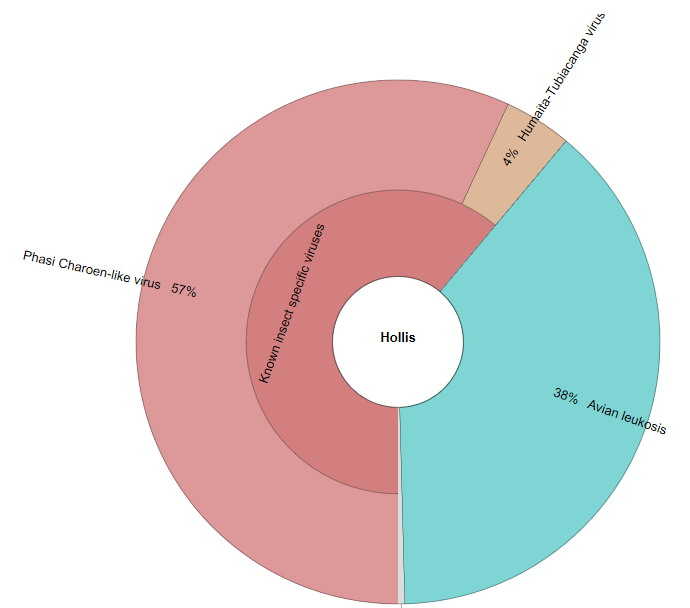


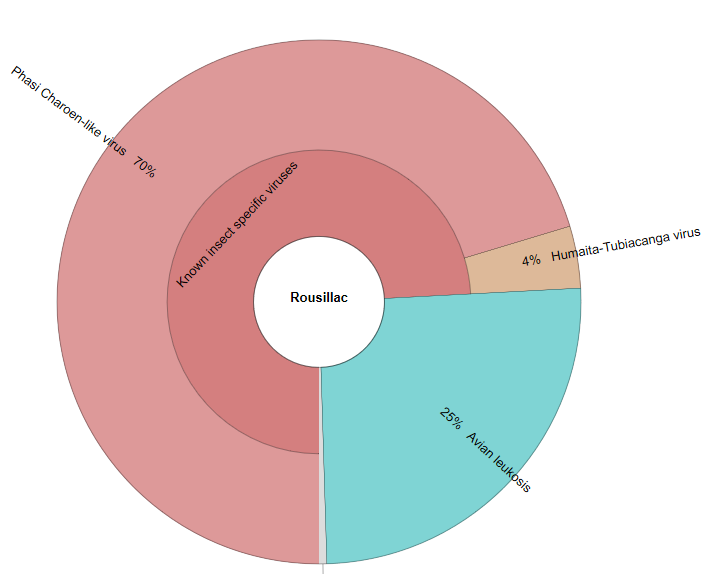

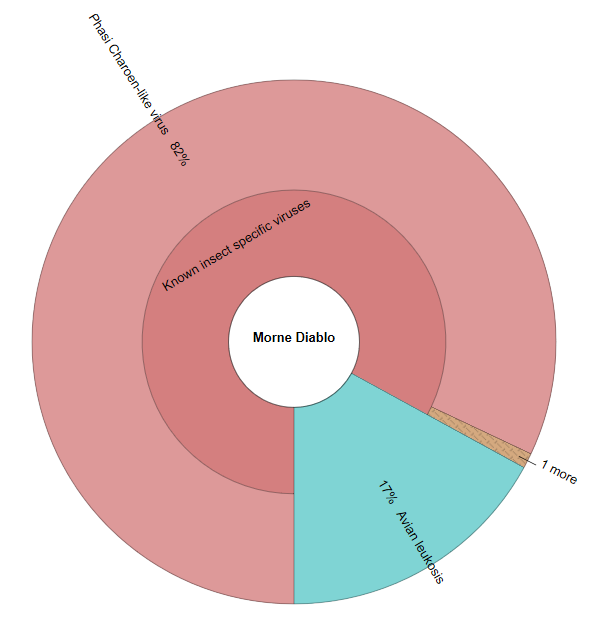


Fig. S2. Metaviromes of *Haemagogus* mosquitos collected from ten (10) forested locations in Trinidad. The Krona charts are based on viral reads mapped onto genomes of major viral groups. n =13,751 for Ecclesville, n=6680 for Mamon village, n=1421 for Chaguaramas, n=123 for Caroni Swamp, n= 159 for Claxton Bay, n=317 for Cumana, n=238 for Hollis, n= 2,063 264 for Mamoral, n=534 for Morne Diablo and n= 208 for Rousillac. There are no Krona visualizations for the (i) Quinam location: only 69 reads aligned the insect associated Black Queen cell virus (ii) Catshill: no viral reads

Fig. S2. Metaviromes for the *Haemagogus* mosquito vector collected from six (6) forested locations in Trinidad. The Krona charts are based on viral reads mapped onto genomes of major viral groups. n =13 751 for Claxton Bay, n=317 for Cumana, n=238 for Hollis, n= 2 063 264 for Mamoral, n=534 for Morne Diablo and n= 208 for Rousillac. There are no Krona visualizations for the (i) Quinam location: only 69 reads aligned the insect associated Black Queen cell virus (ii) Catshill: no viral reads


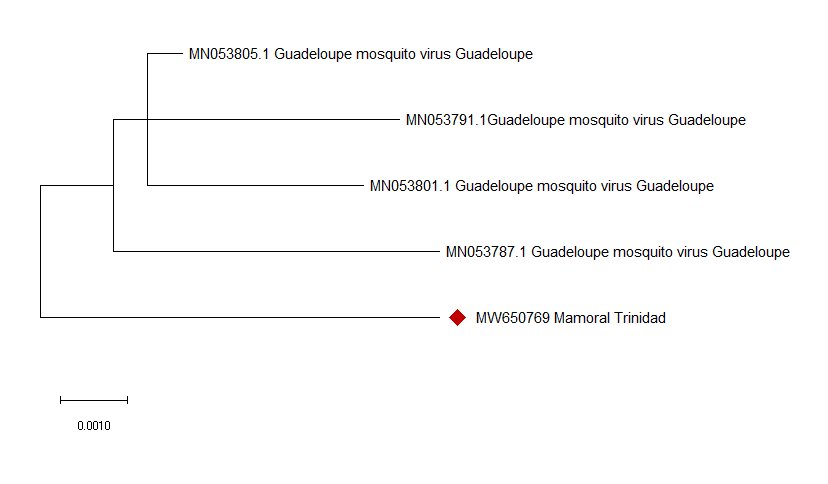

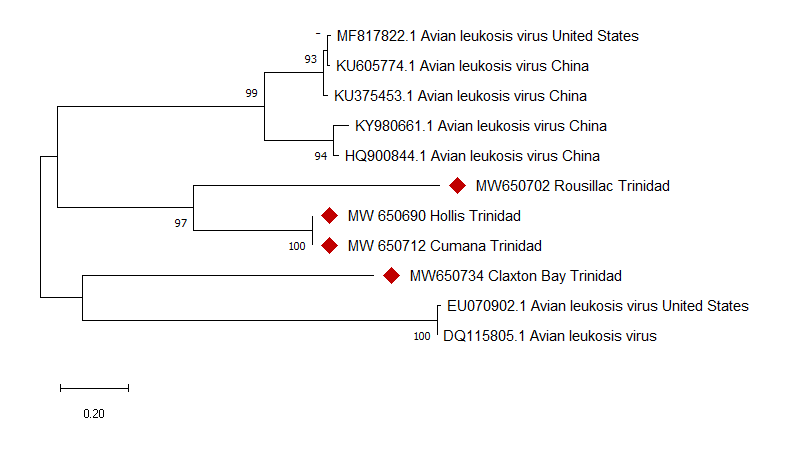

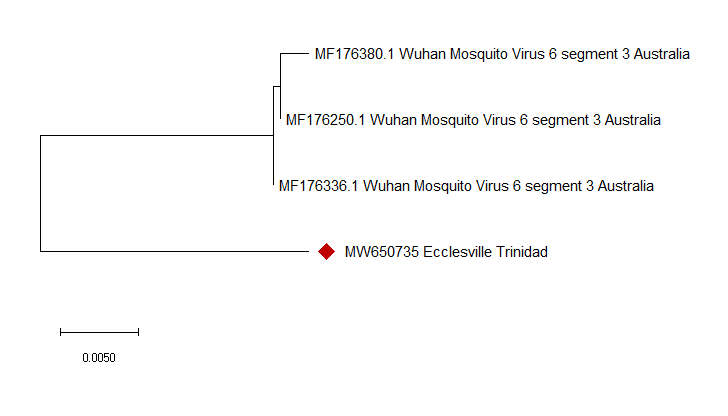


B

A

C


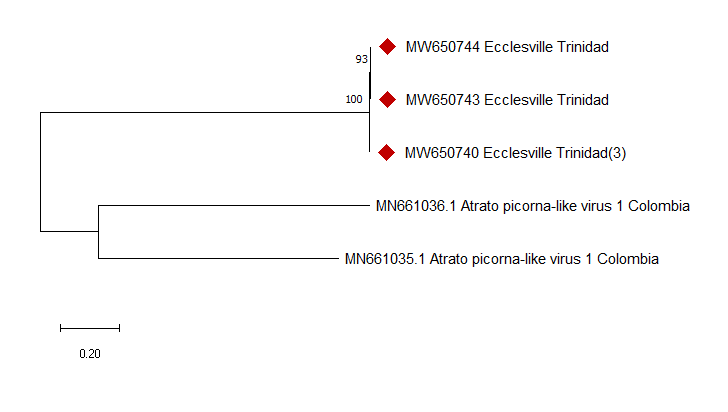


D

Figure S3. Maximum likelihood phylogeny of viral contig sequences found associated with *Haemagogus* mosquitoes in Trinidad (a) Wuhan mosquito virus 6 (segment 3) (b) Guadeloupe mosquito virus (c) new Ecclessville picorna-like virus (d) Avian leukosis virus. Reference sequences were from Genebank and accession numbers are prefixes on the names of the viruses. The trees were constructed in MEGA X (Version 10.1), using the Kimura 2-parameter substitution model and 1000 bootstrap pseudoreplicates. Only bootstrap values ≥ 80% are shown.

Table S1. Identification of viral contigs obtained by mapping and assembly of RNA sequences of *Haemagogus* mosquitoes collected from 12 locations in Trinidad.

| Classification | Type | Virus | Known host | Gene/region | GenBank^a^ Accession No. | Contig length (bp) | Nucleic acid % identity | Location* |
| --- | --- | --- | --- | --- | --- | --- | --- | --- |
| Genus; Phlebovirus ssRNA(−) | Known insect specific viruses | Phasi Charoen-like virus | *Aedes aegypti* | segment M | MW650695  MW650696  MW650697  MW650698  MW650699 | 503  336  801  520  529 | 99.60  99.70  97.63  99.02  99.87 | Hollis |
| Unclassified ssRNA(+) |  | Humaita-Tubiacanga virus | *Aedes aegypti* | RNA dependent RNA polymerase replicase gene | MW650694  MW727415 | 321  249 | 98.44  98.76 |  |
| Genus: Alpharetrovirus  ssRNA(RT) | Other viruses | Avian leukosis virus | Birds | gag polyprotein | MW650689  MW650690  MW650691  MW650692  MW650693 | 809  867  487  467  398 | 99.63  99.88  99.79  99.79  100.00 |  |
| Genus; Phlebovirus ssRNA(−) | Known insect specific viruses | Phasi Charoen-like virus | *Aedes aegypti* | segment S  segment L  segment M | MW650704  MW727416  MW650705  MW650706  MW650707  MW727417  MW650708  MW650709  MW727418  MW650710 | 791 503 336 801 520 529  556  234  659  351 | 98.74  98.81  97.62  97.63 99.62  98.68  98.92  99.15  98.79  98.58 | Cumana |
| Unclassified ssRNA(+) |  | Humaita-Tubiacanga virus | *Aedes aegypti* | RNA dependent RNA polymerase replicase gene | MW773212  MW773213 | 321  243 | 98.44  98.76 |  |
| Genus: Alpharetrovirus  ssRNA(RT) | Other viruses | Avian leukosis virus | Birds | gag protein, polymerase | MW650711  MW650712  MW650713  MW650714  MW650715 | 398  867  467  809  487 | 100.00 99.88 99.79 99.63  99.79 |  |
| Genus; Phlebovirus ssRNA(−) | Known insect specific viruses | Phasi Charoen-like virus | *Aedes aegypti* | segment M  segment L  segment S | MW650780  MW650781  MW650782  MW727425  MW650783  MW650784  MW650785  MW650786  MW650787 | 385  325  571  246  328  459  417  484  781 | 99.48  98.46  99.82  99.19  97.82  98.47  95.44  97.31  98.85 | Caroni bird sanctuary |
| Unclassified ssRNA(+) |  | Humaita-Tubiacanga virus | *Aedes aegypti* | RNA dependent RNA polymerase replicase gene | MW650778  MW650779 | 331  428 | 99.09  99.13 |  |
| Family; Togaviridae Alphavirus ssRNA(+) | Mosquito borne viruses | Mayaro virus | *Haemagogus spp* | nsp1-3, nsp4  structural polyprotein (E3, E2,6K, E1) | M_MAYVF1^b^ M_MAYVR1^b^  M_MAYVF2^b^  M_MAYVR2^b^ | 149  149  150  150 | 100.00  100.00  100.00  100.00 | Mamon village |
| Genus; Phlebovirus ssRNA(−) | Known insect specific viruses | Phasi Charoen-like virus | *Aedes aegypti* | segment M  segment L  segment S | MW650716  MW650717  MW650718 | 3721  6594  792 | 98.09  98.06  98.86 |  |
| Unclassified ssRNA(+) |  | Humaita-Tubiacanga virus | *Aedes aegypti* | RNA dependent RNA polymerase replicase gene | MW650719  MW650720  MW650721 | 426  423  870 | 98.36  97.40  98.04 |  |
| Genus: Alpharetrovirus  ssRNA(RT) | Other viruses | Avian leukosis virus | Birds | gag polyprotein | MW650722  MW650723  MW650724  MW650725  MW650726  MW773205 | 502  1020  427  545  1014  370 | 100.00  99.90  99.30  99.27  99.61  99.73 |  |
| Genus; Phlebovirus ssRNA(−) | Known insect specific viruses | Phasi Charoen-like virus | *Aedes aegypti* | segment M  segment L  segment S | MW650770  MW650771  MW650772 | 3736  6675  830 | 98.10  98.01  98.67 | Mamoral |
| Unclassified ssRNA(+) |  | Humaita-Tubiacanga virus | Aedes aegypti | RNA dependent RNA polymerase replicase gene | MW650773 | 1998 | 97.80 |  |
| Unclassified | Mosquito associated viruses | Guadeloupe mosquito virus strain | *Aedes aegypti Culex quinquefasciatus* | Hypothetical protein  RNA dependent RNA polymerase | MW650769 | 2978 | 99.09 |  |
| Family; Togaviridae Alphavirus ssRNA(+) | Mosquito borne viruses | Mayaro virus | *Haemagogus spp* | nsp1-3, nsp4  structural polyprotein (E3, E2,6K, E1) | E_MAYV1^b^  E_MAYV2 ^b^  E_MAYV3^b^ | 251  277  166 | 99.20  99.28  100.00 | Ecclesville |
| Genus; Phlebovirus ssRNA(−) | Known insect specific viruses | Phasi Charoen-like virus | *Aedes aegypti* | segment M  segment L  segment S | MW650757  MW650758  MW650759  MW650760  MW650761  MW650762  MW650763  MW650764  MW727423  MW650765  MW650766  MW650767  MW650768 | 2001  867  744  476  573  1579  472  792  273  579  312  549  790 | 98.15  98.50  98.66  98.11  97.90  98.35  98.52  97.60  98.90  98.19  99.36  98.72  97.73 |  |
| Unclassified ssRNA(+) |  | Humaita-Tubiacanga virus | *Aedes aegypti* | RNA dependent RNA polymerase replicase gene | MW650739 | 256 | 96.85 |  |
| Family; Bunyaviridae  Phasmavirus like. ssRNA(−) | Mosquito associated viruses | Wuhan mosquito virus 6 | Culex pools | Segment 3 | MW650735 | 2162 | 96.85 | Ecclesville |
| Unclassified Picornavirales |  | Ecclesville picorna like virus | *Haemagogus spp* | polyprotein | MW650740  MW650741  MW650742  MW650743  MW650744 | 4515  4519  320  2134  652 | 96.94  96.77  95.27  96.07  94.94 |  |
| Family Rhabdoviridae  ssRNA(−) |  | Merida virus | *Culex & Ochlerotatus spp* | RNA dependent RNA polymerase | MW650745  MW650746  MW650747  MW650748  MW650749  MW650750  MW650751  MW727420  MW773207  MW773208 | 474  483  602  324  671  528  315  234  294  279 | 98.32  98.96  98.17  99.38  99.11  98.67  97.14  99.15  98.98  97.51 |  |
| Unclassified |  | Hubei virga-like virus 2 | Culicidae mosquitoes | polyprotein | MW650736  MW650737  MW650738 | 5596  3559  547 | 99.14  99.38  99.63 |  |
| Genus: Alpharetrovirus  ssRNA(RT) | Other viruses | Avian leukosis virus | Birds | gag polyprotein | MW727421  MW650755  MW650756 | 277  388  848 | 100.00  99.74  99.76 |  |
| Unclassified |  | Bat Luteovirus | Bats | capsid protein | MW650752  MW650753 | 441  442 | 96.37  95.46 |  |
| Unclassified |  | Bat Sobemovirus | Bats | capsid protein | MW727421  MW650754 | 289  321 | 93.79  95.36 |  |
|  |  |  |  |  |  |  |  |  |
| Genus; Phlebovirus ssRNA(−) | Known insect specific viruses | Phasi Charoen-like virus | *Aedes aegypti* | segment M  segment L  segment S | MW650730  MW650731  MW650732 | 3732  6659  829 | 98.10  97.93  98.55 | Claxton Bay |
| Unclassified | Mosquito associated viruses | Kaiowa virus | *Culex & Stegomiya spp* | Glycoprotein | MW650728 | 826 | 100.00 |  |
| Unclassified |  | Guato virus | *Culex* spp | hypothetical glycoprotein | MW650727 | 818 | 99.81 |  |
| Genus: Alpharetrovirus ssRNA(RT) | Other viruses | Avian leukosis virus | Birds | gag protein | MW650733  MW727419  MW650734  MW773206 | 437  217  434  244 | 100.00  100.00  100.00  97.95 |  |
| Genus:Tobamovirus | Plant viruses | Pepper mild mottle virus | *Capsicum* spp. | replicase protein | MW650729 | 269 | 98.88 |  |
| Genus; Phlebovirus ssRNA(−) | Known insect specific viruses | Phasi Charoen-like virus | *Aedes aegypti* | segment M  segment L  segment S | MW650677  MW727412  MW650678  MW650679  MW650680  MW650681  MW650682  MW650683  MW727413  MW650684  MW650685  MW650686  MW650687  MW773204 | 726  243  547  704  332  335  470  1290  288  378  395  2326  327  262 | 98.76  97.53  98.72  98.58  97.59  98.21  98.30  98.37  98.61  98.68  99.24  98.37  99.39  99.24 | Rousillac |
| Unclassified ssRNA(+) |  | Humaita-Tubiacanga virus | *Aedes aegypti* | RNA dependent RNA polymerase replicase gene | MW650688  MW727414 | 321  243 | 98.44  99.18 |  |
| Genus: Alpharetrovirus ssRNA(RT) | Other viruses | Avian leukosis virus | Birds | gag protein | MW650700  MW650701  MW650702  MW650703 | 324  670  777  434 | 100.00  99.38  100.00  99.89 | Rousillac |
| Genus; Phlebovirus ssRNA(−) | Known insect specific viruses | Phasi Charoen-like virus | *Aedes aegypti* | segment M  segment L  segment S | MW650653  MW650654  MW650655  MW727408  MW650656  MW650657  MW650658  MW650659  MW650660  MW650661  MW650662  MW727409  MW650663  MW650664  MW727410  MW650665  MW650666  MW650667  MW650668  MW650669  MW650670  MW650671  MW650672  MW650673  MW650674  MW650675  MW727411 | 470  587  402  258  695  604  425  830  526  1029  505  292  711  1035  752  602  442  593  300  337  400  808  644  1582  453  365  237 | 98.09  98.81  99.25  99.61  98.42  98.42  97.65  98.19  98.48  99.32  99.60  99.32  99.30  97.68  98.80  99.10  98.87  97.64  99.33  99.49  98.89  98.90  99.22  98.54  98.90  98.63  98.63 | Morne Diablo |
| Unclassified ssRNA(+) | Known insect specific viruses | Humaita-Tubiacanga virus | *Aedes aegypti* | RNA dependent RNA polymerase replicase gene | MW650676 | 1993 | 97.96 | Morne Diablo |
| Genus: Alpharetrovirus ssRNA(RT) | Other viruses | Avian leukosis virus | Birds | gag protein | MW779452  MW779453  MW779454  MW779455  MW779456 MW779457 | 438  300  289  414  625  741 | 99.54  100.00  100.00  96.17  99.52  99.20 |  |
| Genus: Cripavirus ssRNA(+) | Other insect viruses | Black queen cell virus | *Apis* spp | capsid protein | MW650774  MW727424  MW650775  MW650776  MW650777 | 302  208  342  670  461 | 96.03  97.14  96.78  96.12  96.96 | Quinam |
| Unclassified | Mosquito associated viruses | Kaiowa virus | *Culex & Stegomiya spp.* | glycoprotein  partial cds | MW650788 | 1172 | 99.49 | Chaguaramas |
| Unclassified |  | Cumbaru virus | Culicidae *mosquitoes* | glycoprotein  partial cds | MW650789 | 203 | 93.10 |  |
| Unclassified |  | Guato virus | *Culex spp.* | putative  glycoprotein  partial cds | MW650790  MW773209  MW773210  MW773211 | 300  285  256  294 | 98.63  99.33  98.68  99.34 |  |

* No viral reads were detected in mosquitoes from Catshill

a – Contig sequences are available at <https://zenodo.org/record/4932469#.YMQbQKhKi01>

b – not submitted to Genbank

**Table S2.** Identification of contigs generated from reads mapped to mitochondrial sequences of potential blood-meal animal hosts.

| Location | Chordate | Reference GenBank  Sequence of closest match | Contig ID* | Contig length (bp) | Nucleic acid ID% |
| --- | --- | --- | --- | --- | --- |
| Catshill | *Mus musculus* | KY018919.1 KY018919.1 KY018919.1 KY018919.1 KY018919.1  LC062020.1 KY018919.1 KY018919.1 KY018919.1 LC062002.1 KY018919.1 KY018919.1 KY018919.1 KY018919.1 | CAMM1  CAMM2  CAMM3  CAMM4  CAMM5  CAMM6  CAMM7  CAMM8  CAMM9  CAMM10  CAMM11  CAMM12  CAMM13  CAMM14 | 240  610  292  296  320  312  298  279  614  692  831  966  538  509 | 100  100  100  100  100  100  100  100  99.35  100  99.88  100  100  100 |
| Chaguaramas | *Mus musculus* | LC062002.1 LC062002.1  KY018919.1  KY018919.1  KY018919.1  KY018919.1  KY018919.1  KY018919.1  KY018919.1  KY018919.1  KY018919.1 | CHMM1  CHMM2  CHMM3  CHMM4  CHMM5  CHMM6  CHMM7  CHMM8  CHMM9  CHMM10  CHMM11 | 774  496  779  864  1203  1109  688  347  955  420  1550 | 100  100  99.74  100  100  100  100  100  99.89  100  100 |
| Claxton Bay | *Gallus gallus* | MN013407.1 MK163565.1 MK163565.1  KP269069.1 | CBGG1  CBGG2  CBGG3  CBGG4 | 431  326  386  263 | 100  100  100  100 |
| Mamon Village | Gallus gallus | MN013407.1 MK163565.1 MK163565.1 MK163565.1 MK163565.1 MK163565.1 | MAMGG1  MAMBB2  MAMGG3  MAMGG4  MAMGG5  MAMGG6 | 428  318  609  337  303  982 | 100  100  100  100  100  100 |
| Rousillac | *Gallus gallus* | MK163563.1 MK163565.1 MK163561.1 | RGG1  RGG2  RGG3 | 623  203  214 | 100  100  100 |
| Hollis | *Gallus gallus* | MK163565.1 MK163561.1 MK163561.1 MK163561.1 | HGG1  HGG2  HGG3  HGG4 | 347  406  203  491 | 100  100  100  100 |

*** Avian contigs** were assembled from 41, 32, 121 and 48 reads from Claxton Bay, Rousillac, Mamon Village and Hollis locations, respectively, that mapped to *Gallus gallus* mitochondrial genome sequence. Rodent contigs were assembled from1556 and 245 reads from Chaguaramas and Catshill locations, respectively, that mapped to *Mus musculus* mitochondrial sequences.

No reads mapped against mitochondrial sequences of 78 additional bird species, 10 reptile species, and 26 mammals.

Sequences of assembled contigs are available at <https://zenodo.org/record/4932469#.YMQbQKhKi01>.
